# Efficacy of a Hypotonic Treatment for Peritoneal Dissemination from Gastric Cancer Cells: An *In Vivo* Evaluation

**DOI:** 10.1155/2014/707089

**Published:** 2014-07-02

**Authors:** Atsushi Shiozaki, Daisuke Ichikawa, Kenichi Takemoto, Yoshito Nako, Shingo Nakashima, Hiroki Shimizu, Maki Kitagawa, Toshiyuki Kosuga, Hirotaka Konishi, Shuhei Komatsu, Hitoshi Fujiwara, Kazuma Okamoto, Yoshinori Marunaka, Eigo Otsuji

**Affiliations:** ^1^Division of Digestive Surgery, Department of Surgery, Kyoto Prefectural University of Medicine, 465 Kajii-cho, Kamigyo-ku, Kyoto 602-8566, Japan; ^2^Departments of Molecular Cell Physiology and Bio-Ionomics, Graduate School of Medical Science, Kyoto Prefectural University of Medicine, Kyoto 602-8566, Japan; ^3^Japan Institute for Food Education and Health, St. Agnes' University, Kyoto 602-8013, Japan

## Abstract

The aim of the present study was to determine the efficacy of a hypotonic treatment for peritoneal dissemination from gastric cancer cells using an* in vivo* model. We firstly evaluated the toxicity of a peritoneal injection of distilled water (DW) (2 mL for 3 days) in mice. Macroscopic and microscopic examinations revealed that the peritoneal injection of DW did not severely damage the abdominal organs of these mice. MKN45 gastric cancer cells preincubated with NaCl buffer or DW for 20 minutes* in vitro* were then intraperitoneally injected into nude mice, and the development of dissemination nodules was analyzed. The total number, weight, and volume of the dissemination nodules were significantly decreased by the DW preincubation. We then determined whether the peritoneal injection of DW inhibited the establishment of peritoneal dissemination. After a peritoneal injection of MKN45 cells into nude mice, NaCl buffer or DW was injected into the abdominal cavity for 3 days. The total volume of dissemination nodules was significantly lower in DW-injected mice than in NaCl-injected mice. In conclusion, we demonstrated the safeness of a peritoneal injection of DW. Furthermore, the development of dissemination nodules from gastric cancer cells was prevented by a preincubation with or peritoneal injection of DW.

## 1. Introduction

Gastric cancer is a leading cause of cancer-related deaths worldwide, and peritoneal dissemination is the most common form of recurrence in patients with gastric cancer [1, 2]. Peritoneal metastasis is associated with a poor prognosis and, therefore, the management of dissemination in the peritoneal cavity is important in the treatment of gastric cancer [3, 4]. However, there is currently no effective treatment for peritoneal dissemination from gastric cancer. On the other hand, the roles of ion and water channels/transporters have recently been examined in cancer cells, and cellular physiological approaches are expected as novel therapeutic strategies [5–9]. The regulation of extracellular osmolality is a promising method, with previous studies demonstrating the cytocidal effects of hypotonic treatments on cancer cells [10–12]. We recently examined changes in the cellular morphology and volume of gastric cancer cells subjected to hypotonic shock using several unique methods and apparatus, such as a differential interference contrast microscope connected to a high-speed digital video camera and a high-resolution flow cytometer [13]. Our findings confirmed the cytocidal effects of hypotonic shock on gastric cancer cells* in vitro* [13]. However, the effects of hypotonic treatments on the development of peritoneal dissemination from gastric cancer and their safeness* in vivo* have not yet been fully evaluated.

In the present study, we determined, using an* in vivo* model, the toxicity and therapeutic effects of a peritoneal injection of distilled water (DW) for the treatment of peritoneal dissemination from gastric cancer. We showed that the peritoneal injection of DW was not toxic to mice. Furthermore, the development of dissemination nodules from gastric cancer cells was prevented by a preincubation with or peritoneal injection of DW. These results support the efficacies of peritoneal lavage with DW during surgery and the peritoneal injection of DW against dissemination from gastric cancer.

## 2. Materials and Methods

### 2.1. Cell Culture and Materials

The poorly differentiated human gastric adenocarcinoma cell line MKN45 was used in the present study. Cells were grown in plastic culture flasks (Corning Incorporated, NY, USA) and maintained in RPMI-1640 medium (Nacalai Tesque, Kyoto, Japan) supplemented with 10% fetal bovine serum (FBS), 100 U/mL of penicillin, and 100 *μ*g/mL of streptomycin. The flasks were kept in a humidified incubator at 37°C under 5.0% CO_2_ in air.

The 140 mM isotonic NaCl solution (NaCl buffer) contained 140 mM NaCl, 5.0 mM KCl, 1.0 mM CaCl_2_, 1.0 mM MgCl_2_, 5.0 mM glucose, and 10 mM HEPES.

### 2.2. *In Vivo* Experiments

Four-week-old female BALB/c mice were used to evaluate the toxicity of the peritoneal injection of DW. Four-week-old female BALB/c nude mice were used as the peritoneal dissemination model and were purchased from SHIMIZU Laboratory Supplies Co., Ltd. (Kyoto, Japan) and maintained under pathogen-free barrier conditions. Mice were provided with sterile food and water and housed in cages. Ambient light was controlled to provide regular 12 h light-dark cycles. All animal protocols were approved by the institutional guidelines of the Kyoto Prefectural University of Medicine, Kyoto, Japan.

**Figure 1:**
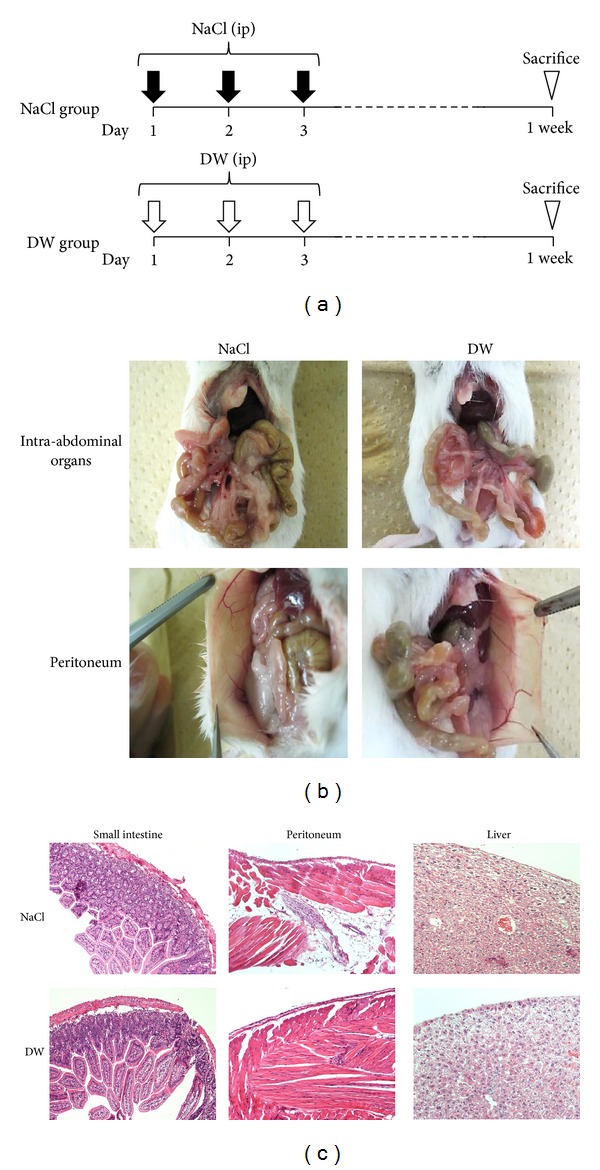
Evaluation of the toxicity of the peritoneal injection of DW. (a) Two milliliters of NaCl buffer or DW was injected into the abdominal cavities of 4-week-old female BALB/c mice for 3 days. At a defined time point of 1 week after the start of the peritoneal injections, all mice were sacrificed, and intra-abdominal findings were investigated. *n* = 3. (b) No significant differences were observed in the representative macroscopic findings of abdominal organs, including the gastrointestinal tract, liver, and peritoneum, between the NaCl and DW groups. (c) No significant differences were observed in the representative histopathological findings of the small intestine, peritoneum, or liver between the NaCl and DW groups. Magnification: 100x. DW: distilled water.

To evaluate the toxicity of the peritoneal injection of DW, 2 mL of NaCl buffer or DW was injected into the abdominal cavities of 4-week-old female BALB/c mice (*n* = 3, each group) for 3 days (Figure 1(a)). The volume of buffer was decided, referring to the circulating blood volume of mouse. At a defined time point of 1 week after the start of the peritoneal injections, all mice were sacrificed, and intra-abdominal findings were investigated. Abdominal organs, including the small intestine, peritoneum, and liver, were fixed in 10% formaldehyde in PBS, paraffin embedded, and stained with hematoxylin and eosin.

**Figure 2:**
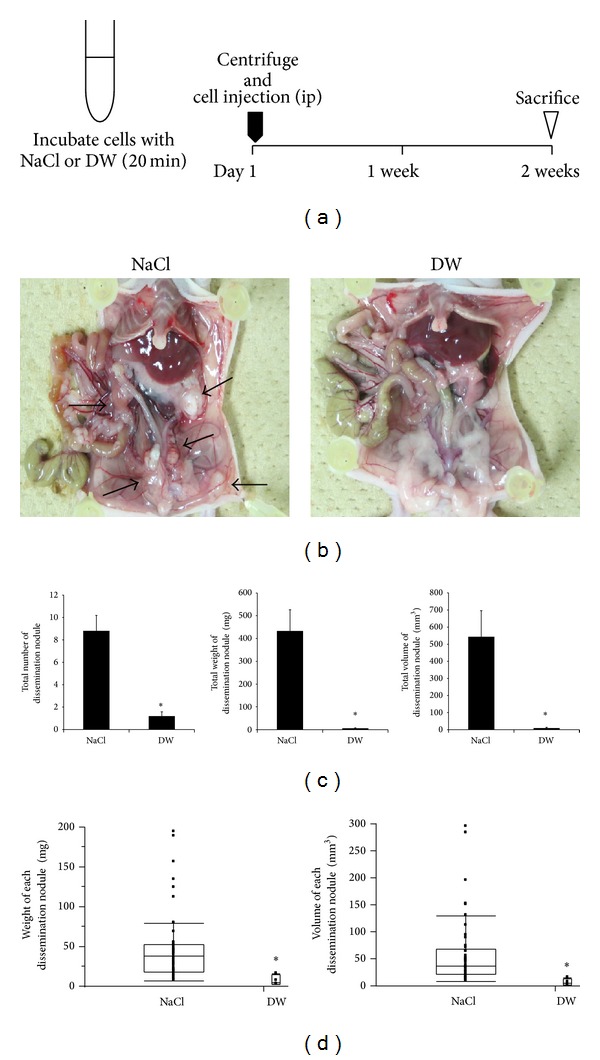
Inhibition of the establishment of peritoneal dissemination after the preincubation of gastric cancer cells with DW. (a) A total of 1.0 × 10^6^ pelleted MKN45 cells were suspended in 5 mL NaCl buffer or DW, and each cell was incubated for 20 min. Thereafter, the suspension was centrifuged, and pelleted cells were suspended in 0.3 mL PBS and then intraperitoneally injected into 4-week-old female nude mice. At a defined time point of 2 weeks after the intraperitoneal injection of cells, all mice were sacrificed, and the degree of peritoneal dissemination was evaluated macroscopically. *n* = 5. (b) Representative macroscopic findings of the abdominal cavity. Many dissemination nodules were established in NaCl preincubated mice, whereas very few tumors were observed in DW preincubated mice. Arrows indicate dissemination nodules. (c) The total number, weight, and volume of dissemination nodules were significantly decreased by the preincubation with DW. The results are presented as the mean ± SEM (*n* = 5)  *P < 0.05. (d) Box plots of dissemination nodules in both groups. The weight and volume of each dissemination nodule were markedly lower in the DW group than in the NaCl group. *P < 0.05. DW: distilled water.

To examine the establishment of peritoneal dissemination from gastric cancer cells exposed to hypotonic shock, MKN45 cells grown in culture flasks were detached and centrifuged. A total of 1.0 × 10^6^ pelleted cells were then suspended in 5 mL DW or isotonic NaCl buffer as a control, and each cell was incubated for 20 min. The incubation time was decided, referring to our previous report [13]. Thereafter, the suspension was centrifuged, and pelleted cells were suspended in 0.3 mL PBS and then intraperitoneally injected into 4-week-old female nude mice (*n* = 5, each group) (Figure 2(a)). All mice were sacrificed at a defined time point of 2 weeks after the intraperitoneal injection of MKN45 cells, and the degree of peritoneal dissemination was evaluated macroscopically. Tumors more than 0.5 mm in diameter were resected and counted, and the weights of the resected tumors were measured. Tumor diameters were measured with a caliper and tumor volumes were calculated using the formula [14]:

(1) $$\begin{array}{c} \text{Tumor}\;\text{volume}=\text{length}\times {\text{width}}^{2}\times 0.5. \end{array}$$

**Figure 3:**
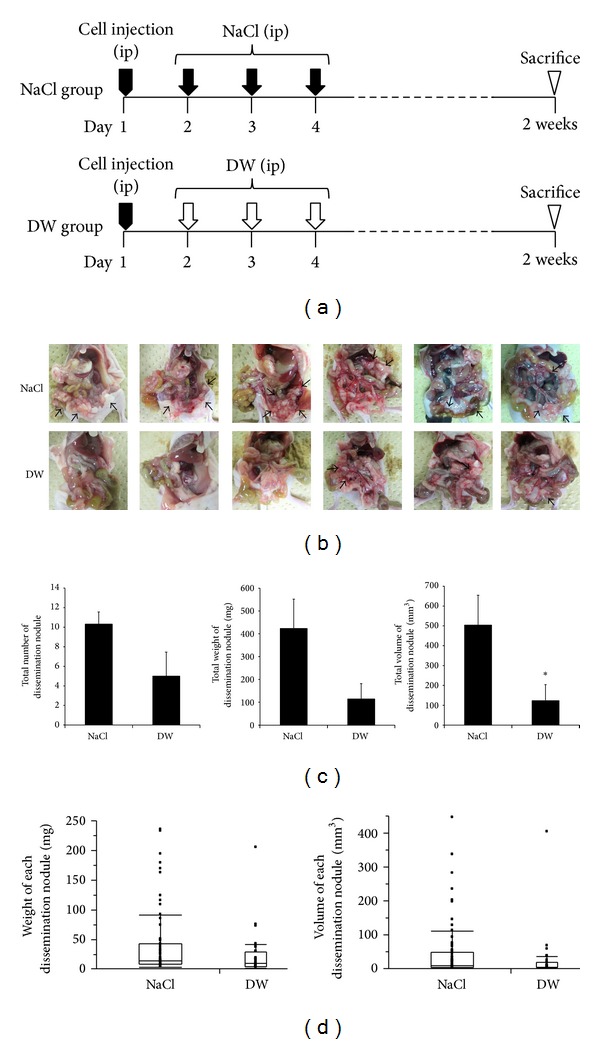
Inhibition of the establishment of peritoneal dissemination by the peritoneal injection of DW. (a) A total of 1.0 × 10^6^ pelleted MKN45 cells were suspended in 0.3 mL PBS and then intraperitoneally injected into 4-week-old female nude mice on day 1. Two milliliters of NaCl buffer or DW was then injected into the abdominal cavities of nude mice from day 2 to day 4 (for 3 days). At a defined time point of 2 weeks after the intraperitoneal injection of cells, all mice were sacrificed, and the degree of peritoneal dissemination was evaluated macroscopically. *n* = 6. (b) Representative macroscopic findings of the abdominal cavities of all mice. Many dissemination nodules were established in all 6 mice injected with NaCl, whereas several nodules were only found in 3 mice injected with DW. Arrows indicate dissemination nodules. (c) The total number, weight, and volume of dissemination nodules in both groups. The total volume of dissemination nodules was significantly lower in the DW group than in the NaCl group. The results are presented as the mean ± SEM (*n* = 6)  *P < 0.05. (d) Box plots of dissemination nodules in both groups. No significant differences were observed in the weight or volume of each dissemination nodule in the two groups. *P < 0.05. DW: distilled water.

To examine the efficacy of the peritoneal injection of DW on the establishment of peritoneal dissemination, MKN45 cells grown in culture flasks were detached and centrifuged. A total of 1.0 × 10^6^ pelleted cells were suspended in 0.3 mL PBS and then intraperitoneally injected into 4-week-old female nude mice (*n* = 6, each group) on day 1. Two milliliters of NaCl buffer or DW was injected into the abdominal cavities of nude mice from day 2 to day 4 (for 3 days) (Figure 3(a)). All mice were sacrificed at a defined time point of 2 weeks after the intraperitoneal injection of MKN45 cells, and the degree of peritoneal dissemination was evaluated macroscopically. Tumors more than 0.5 mm in diameter were resected and counted, and their weights and volumes were analyzed.

### 2.3. Statistical Analysis

Statistical analysis was carried out using Student's* t*-test. Differences were considered significant when the *P* value was <0.05. Statistical analyses were performed using JMP ver. 5 from SAS in Cary, NC, USA.

## 3. Results

### 3.1. Evaluation of the Toxicity of the Peritoneal Injection of DW

To determine the toxicity of the peritoneal injection of DW* in vivo*, 2 mL of NaCl buffer or DW was injected into the abdominal cavities of 4-week-old female BALB/c mice for 3 days (Figure 1(a)). All mice in both groups were alive (*n* = 3, each group), and no abnormal findings were detected following the peritoneal injections. All mice were sacrificed 1 week after the start of the peritoneal injections and intra-abdominal findings were investigated. No significant differences were observed in the macroscopic findings of abdominal organs, including the gastrointestinal tract, liver, and peritoneum, between the NaCl and DW groups (Figure 1(b)). The microscopic findings of the small intestine, peritoneum, and liver revealed no histological damage or inflammation by DW injection or NaCl injection (Figure 1(c)). These results suggested that the peritoneal injection of DW for 3 days did not cause severe toxicity in mice.

### 3.2. Inhibition of Establishment of Peritoneal Dissemination after Preincubation of Gastric Cancer Cells with DW

We previously reported the cytocidal effects of hypotonic shock induced by DW on MKN45 cells [13]. To examine these effects in an* in vivo* model, MKN45 cells incubated with NaCl buffer or DW for 20 minutes* in vitro* were intraperitoneally injected into nude mice, and the development of dissemination nodules was analyzed (Figure 2(a)). Many dissemination nodules were established 2 weeks after the intraperitoneal injection of MKN45 cells preincubated with NaCl, whereas very few tumors were observed in mice injected with MKN45 cells preincubated with DW (Figure 2(b)). Data from 5 mice for each group were analyzed (Figure 2(c)). The total number of dissemination nodules was significantly decreased by the DW treatment (NaCl group: 8.8 ± 1.4; DW group: 1.2 ± 0.8; mean ± standard error of the mean (SEM)). The total weight and volume of dissemination nodules were markedly lower in the DW group (5.8 ± 2.7 mg, 9.1 ± 5.2 mm^3^) than in the NaCl group (433.0 ± 93.5 mg, 544.1 ± 151.9 mm^3^). Each dissemination nodule in both groups was analyzed (Figure 2(d)). A total of 44 dissemination nodules were detected in the NaCl group. The weights of these nodules in the NaCl group ranged from 7.2 to 193.8 mg (median: 37.8 mg; mean ± SEM: 49.2 ± 6.6 mg), and their volumes ranged from 9.4 to 295.3 mm^3^ (median: 38.0 mm^3^; mean ± SEM: 61.8 ± 9.9 mm^3^). On the other hand, only 6 dissemination nodules were detected in the DW group. The weights of these nodules in the DW group ranged from 2.9 to 15.6 mg (median: 5.2 mg; mean ± SEM: 7.8 ± 2.5 mg), and their volumes ranged from 2.3 to 15.8 mm^3^ (median: 5.5 mm^3^; mean ± SEM: 7.6 ± 2.4 mm^3^). The weight and volume of each dissemination nodule were markedly lower in the DW group than in the NaCl group. These results suggest that the preincubation of gastric cancer cells with DW may markedly inhibit the development of dissemination nodules in nude mice.

### 3.3. Inhibition of Establishment of Peritoneal Dissemination by the Peritoneal Injection of DW

We examined whether the peritoneal injection of DW inhibited the establishment of peritoneal dissemination. MKN45 cells were intraperitoneally injected into nude mice on day 1, and 2 mL of NaCl buffer or DW was injected into the abdominal cavities of nude mice from day 2 to day 4 (for 3 days) (*n* = 6 in each group). The development of dissemination nodules was analyzed 2 weeks after the cancer cell injection (Figure 3(a)). As shown in Figure 3(b), many dissemination nodules were established in all 6 mice injected with NaCl buffer, whereas several nodules were found in only 3 mice injected with DW. The total number of dissemination nodules was 50% less with the peritoneal injection of DW than with that of NaCl (NaCl group: 10.3 ± 1.2; DW group: 5.0 ± 2.5; *P* = 0.081) (Figure 3(c)). The total weight of dissemination nodules was slightly less in the DW group (114.8 ± 67.2 mg) than in the NaCl group (423.2 ± 128.6 mg) (*P* = 0.059) (Figure 3(c)). The total volume of dissemination nodules was significantly lower in the DW group (123.8 ± 80.0 mm^3^) than in the NaCl group (504.7 ± 150.2 mm^3^) (Figure 3(c)). We then analyzed each dissemination nodule in both groups (Figure 3(d)). A total of 62 dissemination nodules were detected in the NaCl group. The weights of these nodules in the NaCl group ranged from 1.9 to 235.9 mg (median: 13.6 mg; mean ± SEM: 41.0 ± 7.5 mg), and their volumes ranged from 0.8 to 445.5 mm^3^ (median: 8.8 mm^3^; mean ± SEM: 48.8 ± 11.1 mm^3^). Thirty-one dissemination nodules were detected in the DW group. The weights of these nodules ranged from 0.5 to 205.3 mg (median: 9.1 mg; mean±SEM: 22.2 ± 7.0 mg), and their volumes ranged from 0.8 to 405.0 mm^3^ (median: 5.0 mm^3^; mean±SEM: 25.4 ± 13.0 mm^3^). No significant differences were observed in the weight or volume of each dissemination nodule between the two groups (Figure 3(d)). These results suggested the restrictive efficacy of the peritoneal injection of DW in the initial stage of the establishment of peritoneal dissemination from gastric cancer.

## 4. Discussion

Although recent advances in surgical techniques, adjuvant therapy, chemoradiotherapy, and molecular targeted therapy have improved the prognosis of patients with gastric cancer, the long-term outcomes of these patients remain poor, especially for those with advanced disease. Peritoneal dissemination is the most frequently observed pattern of metastasis and recurrence in gastric cancer patients [3, 4]. Although limited success with treatment methods, such as intraperitoneal chemotherapy, has been reported [15, 16], novel strategies for the treatment of peritoneal dissemination from gastric cancer need to be developed to achieve better results. An improvement in the treatment of peritoneal dissemination from gastric cancer depends on a deeper understanding of the molecular mechanisms regulating tumorigenesis and progression of the disease.

Peritoneal dissemination is considered to be caused by free peritoneal cancer cells exfoliated from serosally invasive tumors, and previous studies have reported the cytocidal effects of hypotonic stress on cancer cells [10–12]. We previously investigated changes in the cellular morphology and volume of gastric cancer cells subjected to hypotonic shock using several unique methods and apparatus [13]. Video recordings using a high-speed digital camera demonstrated that hypotonic shock with DW induced swelling and then rupture in MKN28, MKN45, and Kato-III cells. Measurements of cell volume changes using a high-resolution flow cytometer indicated that severe hypotonicity with DW increased the number of broken fragments of these gastric cancer cells within 5 min. Furthermore, we reincubated these cells after they had been exposed to DW and found that the decrease observed in the number of cells in each of the three gastric cancer cell lines depended on the time for which they had been exposed to DW. Similar findings have been reported in esophageal and pancreatic cancer cells [17, 18]. These results suggest that hypotonic shock could be applied for the treatment of dissemination from gastric cancer by using a peritoneal injection of hypotonic solution. However, to the best of our knowledge, the toxicity of a peritoneal injection of hypotonic solution* in vivo *has not yet been examined in detail. In the present study, we analyzed both macroscopic and microscopic findings and found that the peritoneal injection of DW (2 mL, for 3 days) did not cause severe toxicity in mice. Regarding effects on noncancerous normal cells, we previously showed that severe hypotonic shock also induced cell rupture in human fibroblast WI38 cells [18]. However, the pathological findings of the present study revealed that the effect of hypotonic shock did not appear in the single layer peritoneal cells.

Exfoliated cancer cells were detected in the abdominal cavity following the resection of gastric cancer, and several studies have shown that peritoneal lavage fluid cytology is a significant independent prognostic factor in gastric cancer patients [19–21]. Exfoliated cancer cells from the primary tumor may be viable and tumorigenic; therefore, effective peritoneal lavage is clinically important at the time of the initial surgery. Peritoneal lavage with DW has been performed during surgery for various cancers. Lin et al. reported that peritoneal lavage with DW improved the survival rate of patients with spontaneously ruptured hepatocellular carcinoma [10]. Huguet and Keeling described the optimal method for peritoneal lavage with DW during colorectal cancer surgery [11], and Mercill et al. found that exposure to distilled water reduced the number of surviving gastric cells [12]. In the present study, we intraperitoneally injected gastric cancer cells preincubated with DW into nude mice and analyzed the development of dissemination nodules in order to determine the efficacy of peritoneal lavage with DW during surgery. Our results showed that the total number, weight, and volume of dissemination nodules were significantly decreased by the DW preincubation. Furthermore, the weight and volume of each dissemination nodule were markedly lower in the DW pretreated group than in the NaCl pretreated group. These results suggested that peritoneal lavage with DW during surgery for gastric cancer may be effective at disrupting exfoliated cancer cells and preventing the development of dissemination nodules.

We also determined the efficacy of the peritoneal injection of DW for inhibiting the development of peritoneal dissemination nodules* in vivo* as a future treatment for patients with peritoneal dissemination from gastric cancer. Our results showed that the total volume of dissemination nodules was significantly lower in DW-injected mice than in NaCl-injected mice. These results confirmed the efficacy of the peritoneal injection of DW for preventing peritoneal dissemination from gastric cancer. However, its inhibitory effects appeared to be weaker than those observed in the DW preincubation model, as described above. One reason may be the increase in osmolarity by the DW injection due to the contamination of existing intraperitoneal secretions and many types of cells. We previously reported that very severe hypotonicity was needed to disrupt gastric cancer cells into fragments [13], and, therefore, we investigated only DW condition for* in vivo* model instead of different solutions containing different concentration of solute in the present study. We previously showed that the osmolarity of the fluid collected after peritoneal lavage with DW during surgery for gastric cancer was approximately 50 mosmol/kgH_2_O due to the contamination of disrupted cells [13]. To keep the intra-abdominal osmolality as low as possible, the maximal dose of DW needed to be administered to mice. Therefore, we decided to inject 2 mL of buffer, referring to the circulating blood volume of mouse in the present study. The persistent perfusion of the abdominal cavity with DW may overcome this phenomenon and become an effective technique in clinical practice; however, it is difficult to establish an* in vivo* experimental model with mice.

Our results revealed that no significant differences in the weight and volume of each dissemination nodule existed between the DW-injected and NaCl-injected groups, whereas a prominent inhibitory effect was found in the DW preincubated model. These results suggested that the peritoneal injection of DW may not have enough effect for already established dissemination, whereas the cytocidal effects of hypotonic shock were sufficient for isolated single cells. Therefore, the hypotonic solutions used in peritoneal injections should be modified to enhance their inhibitory effects. The roles of ion and water channels/transporters have recently been reported in cancer cells [5–9] and are expected to become novel therapeutic targets. Various types of transporters have been found in gastric cancer, and we previously investigated the roles of Cl^−^ channels/transporters [22–25]. We showed that a treatment with 5-nitro-2-(3-phenylpropylamino) benzoic acid (NPPB), a Cl^−^ channel blocker, increased cell volume by inhibiting regulatory volume decrease (RVD) and enhanced the cytocidal effects of the hypotonic solution in MKN45 cells [13]. RVD was shown to occur after hypotonicity-induced cellular swelling and has been attributed to the activation of ion channels and transporters, which cause K^+^, Cl^−^, and H_2_O effluxes, ultimately leading to cell shrinkage [26, 27]. Similar phenomena have been reported in esophageal and pancreatic cancer cells [17, 18]. We could not use NPPB, which blocks multiple types of chloride channels, in the present study because of its neurotoxicity* in vivo*. However, the development of a more specific chloride ion channel blocker or novel siRNA delivery system* in vivo* will make it possible to enhance the effects of the peritoneal injection of DW in the treatment of dissemination from gastric cancer. Furthermore, a hypotonic intraperitoneal cisplatin treatment with DW at the time of gastric resection was tolerated well by patients with gastric cancer [28], which suggested that the intraperitoneal DW injection may increase the uptake of anticancer drugs and enhances antitumor effects. Our results together with previous findings show the importance and possible application of these cellular physiological approaches for patients with dissemination from gastric cancer.

In conclusion, we demonstrated the safeness of a peritoneal injection of DW in an* in vivo* model. Our results indicated that the development of dissemination nodules* in vivo* was prevented by the preincubation of gastric cancer cells with DW or peritoneal injection of DW. A deeper understanding of the underlying molecular mechanisms may lead to the application of this cellular physiological approach, such as the regulation of osmolality, as a novel therapeutic strategy for dissemination from gastric cancer.
